# Woodland Recovery after Suppression of Deer: Cascade effects for Small Mammals, Wood Mice (*Apodemus sylvaticus*) and Bank Voles (*Myodes glareolus*)

**DOI:** 10.1371/journal.pone.0031404

**Published:** 2012-02-08

**Authors:** Emma R. Bush, Christina D. Buesching, Eleanor M. Slade, David W. Macdonald

**Affiliations:** Wildlife Conservation Research Unit, Department of Zoology, University of Oxford, Oxford, United Kingdom; Northwestern University, United States of America

## Abstract

Over the past century, increases in both density and distribution of deer species in the Northern Hemisphere have resulted in major changes in ground flora and undergrowth vegetation of woodland habitats, and consequentially the animal communities that inhabit them. In this study, we tested whether recovery in the vegetative habitat of a woodland due to effective deer management (from a peak of 0.4–1.5 to <0.17 deer per ha) had translated to the small mammal community as an example of a higher order cascade effect. We compared deer-free exclosures with neighboring open woodland using capture-mark-recapture (CMR) methods to see if the significant difference in bank vole (*Myodes glareolus*) and wood mouse (*Apodemus sylvaticus*) numbers between these environments from 2001–2003 persisted in 2010. Using the multi-state Robust Design method in program MARK we found survival and abundance of both voles and mice to be equivalent between the open woodland and the experimental exclosures with no differences in various metrics of population structure (age structure, sex composition, reproductive activity) and individual fitness (weight), although the vole population showed variation both locally and temporally. This suggests that the vegetative habitat - having passed some threshold of complexity due to lowered deer density - has allowed recovery of the small mammal community, although patch dynamics associated with vegetation complexity still remain. We conclude that the response of small mammal communities to environmental disturbance such as intense browsing pressure can be rapidly reversed once the disturbing agent has been removed and the vegetative habitat is allowed to increase in density and complexity, although we encourage caution, as a source/sink dynamic may emerge between old growth patches and the recently disturbed habitat under harsh conditions.

## Introduction

Increases in range and density of deer populations in the Northern hemisphere (e.g. U.K [1], Continental Europe [2], North America [3]) have been suggested as the prime drivers of changes in woodland vegetation over the past 40 years [4]–[6]. Grazing as a disturbing influence can lead to stalled regeneration, decline in undergrowth cover and altered composition of the ground layer [7]–[9].

In large parts of the UK, these effects are exacerbated by the spread of non-native species, such as fallow deer (*Dama dama* L.), sika (*Cervus nippon*, Temminck), and muntjac (*Muntiacus reevsii*, Ogilby) [10] alongside the native roe (*Capreolus capreolus*, L.) and red deer (*Cervus elaphus*, L.). The ecosystem functionality of deer species differs according to their body size and digestive system [11] as well as their range and habitat requirements. Larger bodied species such as red and roe deer take larger amounts of low quality forage, whereas small deer such as muntjac require smaller amounts of high quality food such as buds, growing shoots, and saplings [10].

The effects of grazing cascade throughout the ecosystem, with particular impacts on animals that rely on dense undercover, for example as a nesting habitat, for provision of food or as protective cover from predators. Thus, many species have shown marked declines associated with increased grazing pressure, including birds [12], [13], woodland invertebrates [14] and small mammals [15].However, in the past, few attempts have been made to observe thresholds above which these higher order processes such as the diversity and abundance of the animal community are affected (but see [16] for invertebrates and small mammals) and their ability to recover once the pressure is removed.

Small mammals, such as bank voles (*Myodes glareolus*, Schreber), and wood mice, (*Apodemus sylvaticus*, L.), play an important role in the dynamics of woodland ecosystems. Their feeding habits implicate them within cycles of vegetative regeneration [17]. As common prey species, they contribute to the diet of many mammalian and avian predators, some of which are of particular conservation concern [18]; for example, weasels (*Mustela nivalis*, L.) primarily prey on small rodents and have a patchy distribution that has been associated with declines in their rodent prey species caused by agricultural intensification [19]. It has therefore been suggested that small mammals can be used as reliable indicator species of ecosystem health [20].

Experimental deer exclosures in Wytham Woods, Oxfordshire, UK, have been used to show empirically that deer have been a causal factor in both vegetative change [8] and in the marked decline in bank vole numbers [21] observed over the past 40 years [15], [22]. The differing effects of deer grazing on the two most common small mammal species – wood mice and bank voles - can be attributed to their respective survival strategies determined by the differing niches they inhabit. Voles feed mostly on herbaceous material and the fleshy parts of the major fruiting species of the shrub layer, such as elder (*Sambucus nigra* L.), blackberry (*Rubus fruticosus* agg.), spindle (*Euonymus europaeus* L.) and hawthorn (*Crataegus monogyna* Jacq.), but do not consume the hard inner testa, instead feeding on the soft testa of various ground dwelling dicotyledons, such as dog's mercury (*Mercurialis perennis* L.), bluebells (*Hyacinthoides non-scripta* L. Chouard ex Rothm) and nettles (*Urtica diocia* L.) [17]. They have also been observed to feed on tree fruits such as that from the European ash (*Fraxinus excelsior* L.) when available [23].They rely on thick groundcover to protect from predation and supply suitable forage [24]. Mice are omnivorous, feeding preferentially on arthropods and seeds, able to consume even the hard testa of fruit kernels such as blackberry (*R. fruticosus*), and canopy tree seeds such as sycamore (*Acer pseudplatanus* L.) [17]. They are agile, allowing escape from predators even in open areas [24] and forage over a greater height distribution amongst the shrub and tree layers [25] allowing them to utilise and establish successful populations in disturbed habitat despite lower levels of cover (see related system of deer mice in burned forest, Montana [26]). It is interpreted that the deer-free exclosures - as an example of patchily distributed bramble thicket- served as high quality habitat for bank voles and allowed a source population to form, enabling dispersal into the surrounding sub-optimal habitat.

Wood mice are nocturnal and usually breed between March and October, with a maximum lifespan of 18–20 months and weight between 13–27 g. Bank voles are diurnal, breeding between April and October with maximum lifespan of 18 months and weight between 14–40 g [27]. In both species, adult males have larger home ranges, overlapping those of several females [28] with a recorded average of 0.63 ha for male vs. 0.19 ha for female mice, and 0.2 ha male vs. 0.14 ha female voles in woodland habitat [27]. Females, in contrast, have exclusive breeding territories, in order to defend food resources and pups from infanticide. Abundance, distribution and renewal rate of food resources as well as season and breeding condition are thought to determine territoriality [29], [30].

Over the past decade, management of Wytham Woods has made a concerted effort to reduce the overall deer numbers via sustained culling; from 0.4–1.6 deer per hectare in late 1990s [21] to <0.17 deer per hectare since 2003 onwards [31]. The vegetative habitat has begun to recover in the open woodland along a trajectory similar to that inside the exclosures, except at a slower rate due to maintained low levels of grazing [6].

Here, we sought to understand the long-term consequences of a period of high deer density by comparing the vegetative habitat and the small mammal community of the deer-free exclosures and surrounding open woodland with the data collected from 2001–2003 [21].

We aimed to address the following hypotheses;

1. That size of bank vole and wood mouse populations within the exclosures would be equivalent to those in the open woodland (in terms of abundance, survival rates and interspecific ratios), without a source/sink dynamic between the two.
2. That various demographic metrics, as a proxy for fitness of individuals, will show no difference between exclosures and open woodland.
3. That the ground flora and undergrowth of the open woodland will show signs of recovery when compared to the exclosures and data from 2001–2003.

## Methods

### 1. Study Site

The study was conducted between 21.06.2010 and 19.11.2010 in Wytham Woods, Oxfordshire, UK (SP 462080; for a detailed description of the site see [31]). Small mammal trapping and vegetation surveys were conducted in three sites - Swinford (SF), Firebreak (FB) and Marley (ML) - located in different parts of the woodland, but all in areas of ancient semi-natural woodland. Each site comprised a deer exclosure (ca. 0.3 ha, roughly rectangular in shape, and protected by a 2 m high deer fence: mesh size 15×15 cm), which were established in 1997 as control plots to investigate the effects of the absence of deer grazing on vegetation structure (for details see [8]). Transects parallel to the perimeter of the exclosures were established 20 m into the open woodland to test the effects of deer grazing and to allow pair-wise comparisons with exclosures.

### 2. Trapping protocol

Each of the three sites was trapped five times with intervals of approximately five weeks between trapping sessions (June, July, September, October, and November) totalling 4500 trap-nights. The trapping regime was planned as to follow that of [21] to allow comparison after a seven year interval of stringent deer control. Fifty Longworth live traps were spaced evenly along the perimeter of the deer fence facing inwards (to minimise vegetation trampling), whilst another 50 traps were set along the four transects to allow site-specific pair-wise comparisons between the exclosure and open woodland at each of the three sites.

For each session, traps were set for three nights and days, and checked twice daily, at dawn and dusk. Traps were filled with hay for bedding, guinea pig muesli and bird seed as food, a slice of carrot to provide moisture, and casters to provide food for accidental captures of shrews [32]. For each capture, species and trap location (exclosure/ open woodland) were recorded, and animals were sexed, weighed, aged (categorised as juvenile: immature pelage, adult: completed adult moult [33]); given an index of reproductive condition (male: non-reproductive: testes fully or partly ascended, reproductive: fully descended testes and/or visible scent gland; female: non-reproductive: imperforate, or no sign of pregnancy, reproductive: pregnant, visible teats or finished lactating) and marked with a unique fur clip for individual identification. For recaptures, the existing clip mark was recorded.

### 3. Vegetation survey

Vegetation surveys were undertaken at each site in June (at the start of the study) and September (to coincide with peak abundance of fruits and seeds) to assess the availability of potential herbivorous food resources as well as protection from predators through the level of cover.The percentage cover and species composition of the canopy (>2.5 m), undergrowth layer (0.5–2.5 m) and ground flora (<0.5 m) were recorded at every fifth trapping point along the inside edge of each of the three exclosures and along each of the four transects at each site, giving a total of 20 replicates at each site, 10 in the exclosure and 10 in the open woodland. The canopy was assessed for openness and tree species composition using a canopy scope [34]. The undergrowth was given a value for overall and constituent species percentage cover in a 1 m×1 m quadrat. In September, the number of fruiting apices for bramble was also recorded. For the ground layer a quadrat was used to assess percentage cover in four categories: vegetation, woody debris, bare soil and leaf litter / moss / twigs. Angiosperms were identified to species and their percentage cover recorded.

### 4. Data analysis

#### Population parameters

Population parameters were derived using the mutli-state Robust Design method with the conditional (Huggins) option [35]–[37] in the software program MARK [38]. The data set consisted of five primary occasions (i.e. five trapping sessions) and three secondary occasions (i.e. the three days over which each site was trapped during the primary occasions). The robust design method can be used to estimate population size (N), encounter probability (p) and recapture probability (c) for each primary occasion using closed capture theory and survival (S), and movement (Ψ^ExOw^, Ψ^OwEx^), between primary occasions using Cormack Jolly-Seber analysis. Two model sets were constructed, one each for bank voles and wood mice. Site was incorporated in the model as an exclusive ‘group’ factor. Each capture or recapture incident was assigned a ‘state’ to identify the environment in which the animal was encountered at a site (exclosure vs. open woodland) and to allow for movement between the two.

Models were compared using the corrected Akaike's Information Criterion (AICc), a measure of the model's likelihood and fit to the given data set taking into account effective sample size. The model with the lowest AICc is the most parsimonious and thus considered the closest to the ‘true’ scenario [39]. As a rule of thumb, a model is deemed a better fit to the data if the difference in AICc between the best fitting model and a competing model (ΔAICc), is equal to or greater than two units [38].

As an initial step the best general model (i.e. most parameterized) was chosen by comparing different scenarios for movement between states; no movement (Ψ^ExOw^ = Ψ^OwEx^ = 0), random movement (Ψ^ExOw = ^Ψ^OwEx^) and markovian movement with standard constraints (Ψ^ExOw^_k_ = Ψ^ExOw^_k−1_ Ψ^OwEx^_k_ = Ψ^OwEx^_k−1_). Then the parameters for survival, movement, encounter, and recapture were allowed to vary both by time and state, and the best model was chosen by comparison of ΔAICc values [39]. All models within two units of the best fitting model were averaged to obtain unbiased parameter estimates [40]. Parameters were compared between environments using the model competitions as described above and unconditional 95% confidence intervals.

Edge effects bias the calculation of population density for all trapping layouts [41] where it is not possible to calculate home range size for the species, which in itself is variable by habitat, individual, and by season. The same number and layout of traps were deployed in the exclosures and the open woodland, and thus we used ‘number of animals’ rather than density as a relative comparison of the suitability of the environments for the species.

Sites were compared for overall abundance of animals using Pearson's chi-squared test.

#### Population Demographics

Metrics for population age structure, adult sex composition and adult reproductive effort (by sex) were constructed as percentages of the minimum number alive (MNA) counts of unique individuals and analysed using the non-parametric Wilcoxon rank sum test to compare between environments within each site. Animals that moved between the environments were excluded from this analysis, to enable characterisation of the permanent population.

Weight, as a proxy for individual fitness was analysed using a GLM accounting for sex, site and habitat. Adult males and non-reproductive adult females were included in the analysis (pregnant females were excluded to avoid bias). If an individual occurred in more than one primary period then an average weight was used in the analysis to maintain independence.

#### Vegetation Survey

Each structural level, Wilcoxon rank sum tests were used to compare exclosure and open woodland vegetation; species diversity of the field layer, percentage cover and composition of the undergrowth, and level of canopy cover. A fruiting index was created for blackberries from the autumn survey by standardising the number of fruiting apices by the percentage cover of bramble within the quadrat to assess the maturity of the vegetation and the availability of a key food resource.

Wilcoxon rank sum tests and chi-squared tests were carried out using the statistical software package PAST 2.04 [42], whilst ANOVAs were carried out using MINITAB 15 [43].

## Results

### 1. Population parameters

Over the study period we recorded a total of 730 captures, of which 365 were bank voles (149 unique individuals), and 365 were wood mice (163 unique individuals).

The outcome of the model competition for the bank vole data set pointed toward a locally and temporally heterogeneous population across sites (FB, SF, ML) and environments (Ex, Ow) (see Table 1A for model competition and Table 2A for mean parameter estimates).

**Table 1:** Outcome of model competitions.

A. Bank Voles				
Mo^1^	Model^2^	AICc^3^	Δ AICc^4^	Par^5^
M	S(site*state*time) Ø_ExOw_(site*state) Ø_OwEx_(site*state) p(site*state*t1*t2) = c	1461.0	0.0	120
M	S(site*state*time) Ø_ExOw_(site*state*time) Ø_OwEx_ (site*state*time) p(site*state*t1*t2) = c	1666.7	205.8	94
R	S(site*state*time) Ø_ExOw_(site*state*time) = Ø_OwEx_ (site*state*time) p(site*state*t1*t2) = c	1721.9	261.0	108
N	S(site*state*time) Ø^ExOw^(0) Ø^OwEx^(0) p(site*state*t1*t2) = c	39700.7	38239.7	116

B. Wood mice				
Mo^1^	Model^2^	AICc^3^	Δ AICc^4^	Par^5^
R	S(site) Ø_ExOw_(site) = Ø_OwEx_(site) p(site*state) = c	1488.1	0.0	12
R	S(site) Ø_ExOw_(site) = Ø_OwEx_(site) p(site) = c	1489.5	1.4	9
R	S(site*state) Ø_ExOw_(site) = Ø_OwEx_(site) p(site*state) = c	1490.0	1.9	15
M	S(site*state*time) Ø_ExOw_(site*state*time) Ø_OwEx_(site*state*t1) p(site*state*t1*t2) = c	1565.4	77.4	132
N	S(site*state*time) Ø_ExOw_(0) Ø_OwEx_(0) p(site*state*t1*t2) = c	21979.7	20491.6	126

1 Mo = type of movement (M = markovian with standard constraints, R = random, N = no movement).

2 Model notation: Parameters, S (survival), Ø_ExOw_ (movement from exclosure to open woodland) Ø_OwEx_ (movement from open woodland to exclosure), c(encounter rate), p (recapture rate). Constraints, site (SF, FB,ML), state (exclosure vs. open woodland), t1 (time between primary periods), t2 (time between secondary periods).

3 AICc = corrected Aikake's Information Criterion.

4 Δ AICc = difference in AICc from best model.

5 Par = number of parameters estimated.

Outcome of model competitions for both species showing fully saturated (most parameterized) general model with different movement options (markovian, random or none), the best constrained model of that movement type (Δ AICc = 0) and all models within two AICc units of this best model. Constraints include site, state (environment) and time. The best model for the bank vole data is markovian, with differential rates of movement between the exclosure and the open woodland. The best model for the wood mice data is random, with equal rates of movement between exclosure and open woodland.

**Table 2:** Mean parameter estimates.

A. Bank Voles		
Parameter	Exclosure	Open Woodland
S1*	0.55 (0.05)	0.49 (0.12)
S2*	0.64 (0.19)	0.66 (0.05)
S3*	0.62 (0.13)	0.47 (0.24)
S4*	0.14 (0.08)	0.16 (0.08)
Ø_ExOw_	0.27 (0.15)	
Ø_OwEx_	0.33 (0.05)	
p1:1**	0.29 (0.15)	0.28 (0.11)
P1:2**	0.12 (0.12)	0.47 (0.26)
p1:3**	0.51 (0.29)	0.34 (0.07)
p2:1**	0.30 (0.15)	0.22 (0.05)
p2:2**	0.67 (0.07)	0.47 (0.08)
p2:3**	0.57 (0.1)	0.61 (0.08)
p3:1**	0.44 (0.08)	0.17 (0.03)
p3:2**	0.51 (0.08)	0.34 (0.13)
p3:3**	0.52 (0.13)	0.58 (0.13)
p4:1**	0.33 (0.09)	0.15 (0.08)
p4:2**	0.42 (0.14)	0.16 (0.08)
p4:3**	0.43 (0.19)	0.36 (0.19)
P5:1**	0.34 (0.08)	0.31 (0.09)
P5:2**	0.44 (0.18)	0.37 (0.09)
P5:3**	0.31 (0.11)	0.25 (0.05)

B. Wood Mice		
Parameter	Exclosure	Open Woodland
S	0.39 (0.04)	0.35 (0.06)
Ø_ExOw_	0.29 (0.08)	
Ø_OwEx_	0.29 (0.08)	
p	0.48 (0.03)	0.41 (0.05)

Parameters: S (survival), Ø (movement between exclosure and open woodland), p (encounter rate).

*Interval between primary periods.

**Number(Primary period): Number(Secondary period).

Model averaged parameter estimates taken from all models within two AICc units of the best model (Table 1.) Means taken from across sites (SF, FB, ML) for exclosure and open woodland environments with standard error shown in brackets. The best model for wood mice has fewer parameters than that for bank voles, mainly because of less variability across the season.

**Figure 1:**
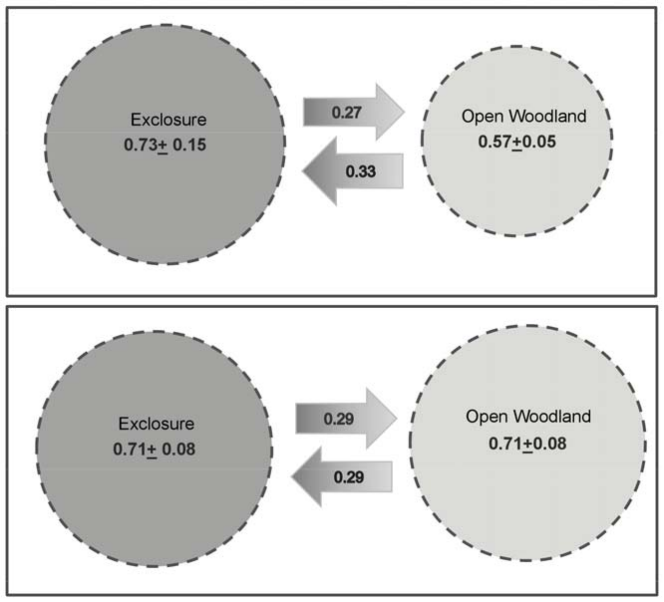
Schematic to show mean probabilities for movement between exclosures and open woodland. Scaled schematic showing probability of staying in same state (exclosure or open woodland) as that in which first encountered (circles) and probability of moving between states dependent on state in which first encountered in (arrows). Values shown are mean model averaged parameter estimates from the three sites with standard error in brackets.

The best fitting model for voles allowed apparent survival to vary by state, but parameter confidence intervals overlapped and there was no particular trend in survival between the two states (Ex vs. Ow). The best model for movement was markovian; probability of movement depended on direction of movement between the exclosures and the open woodland. There was no clear pattern for movement across the sites, but on average if a vole was found in the exclosure it had a higher probability of staying there to the next primary occasion (0.73±0.15), than if it were in the open woodland (0.57±0.05). Meaning that movement into the exclosures from the open woodland was more likely than the opposite movement (Figure 1A). Encounter probability varied for voles by state (Ex vs. Ow) and by time, within both primary and secondary periods. Again there was no clear pattern to this and confidence intervals crossed widely.

**Figure 2:**
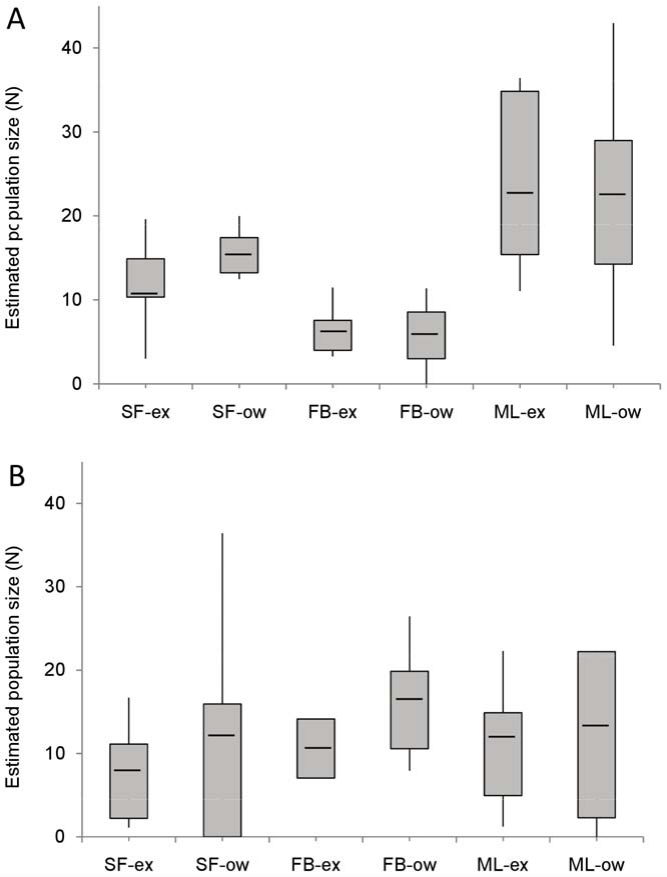
Summary plots of model averaged population size estimates. A. Bank Voles. B. Wood Mice. Sites: Swinford (SF), Firebreak (FB) and Marley (ML), exclosure (-ex), open woodland (-ow). Summary plots showing maximum, upper quartile, mean, lower quartile and minimum values for population size across the trapping season (June–November). The model averaged estimates for population size (N) from the multi-state Robust Design method gave equivalent estimates for deer-free exclosures and open woodland transects at each site. Bank vole numbers varied significantly by site (χ^2^_2_ = 53.73, p<0.001), but wood mice did not (χ^2^_2_ = 3.2, p>0.1).

The estimated number of animals did not vary by environment ; confidence intervals crossed widely with equivalent numbers sustained between exclosures and the open woodland. Using the combined abundance from both environments at each site from the peak period of the season, October, we showed that numbers varied significantly by site (χ^2^_2_ = 53.73, p<0.001, Figure 2A). ML sustained the highest peak for voles (N_max_ = 43) whilst FB sustained the lowest (N_max_ = 11).

The model competition for the wood mouse data set reflected a homogeneous population across sites, the season and between the exclosures and open woodland (see Table 1B for model competition and Table 2B for mean parameter estimates).

Wood mouse survival was not affected by environment; the best model did not allow the parameter for apparent survival to vary by the state the individual was encountered in. Similarly the best model for movement was random, with directionality not a significant factor. Mean probability of staying within the state first encountered was 0.71±0.08se irrelevant of state (Figure 1B). Encounter probability did vary by state but not by time, neither primary nor secondary. Despite this, confidence intervals for this parameter overlapped and the difference between exclosure and open woodland was not significant.

In general abundance estimates did not vary by environment (Figure 2B), although at the peak of the season, in both FB and SF mice in the open woodland outnumbered those in the exclosures by 2.2 and 1.9 respectively, with non-overlapping confidence intervals. This was not maintained throughout the season and not observed at ML. Unlike the voles, there were uniform numbers of mice across the sites. A comparison of the combined abundances at each site at the peak of the season, October, showed they were not significantly different ( χ^2^_2_ = 3.2, p>0.1, Figure 2A).

### 2. Inter-specific differences

**Figure 3:**
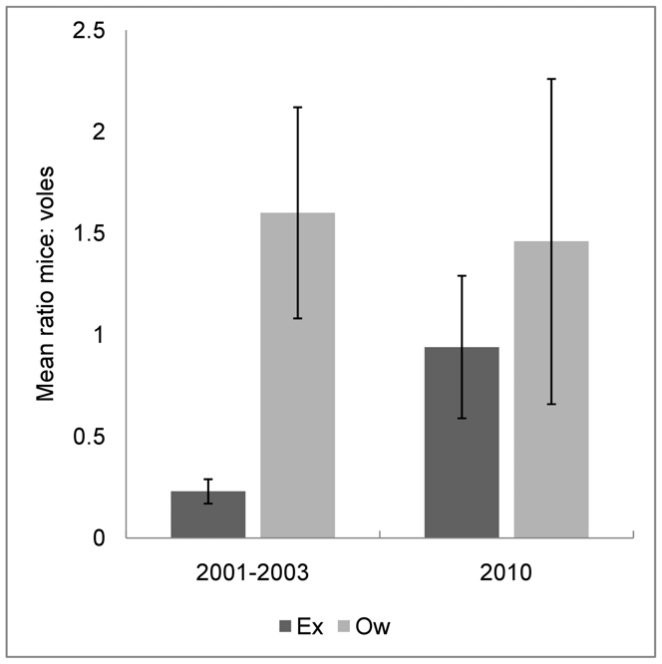
Ratio mice to voles compared for 2001–2003 and 2010 data sets. Environments: Exclosure (Ex), Open woodland (Ow). The data presented here are the mean ratios mice: voles (± standard error) for the deer-free exclosures and the open woodland and show how the small mammal community composition has begun to equalize since deer removal. Data for 2001–2003 courtesy of Buesching *et al.* [21].

On average, wood mice in Wytham outnumbered bank voles by a ratio of 0.94±0.35se in the exclosures and by a ratio of 1.46±0.48se in the open woodland (N = 3, W = 6, p = 0.11, wilcoxon rank sum test, Figure 3). The large standard errors are due to the significant difference in vole numbers between sites but not mice, FB appears as an outlier with much greater numbers of mice than voles compared to SF and ML (see Table 3).

**Table 3:** Interspecific ratios to show relative numbers of wood mice to bank voles across sites.

Environment	SF	FB	ML	Mean (se)
Exclosure	0.70	1.62	0.49	0.94 (0.35)
Open Woodland	0.79	3.06	0.53	1.46 (0.80)

Sites: SF (swinford) FB (Firebreak) ML (Marley).

Mean monthly abundance wood mice / bank voles in order to assess community composition. FB is an outlier with very few voles compared to mice.

### 3. Population demographics

There were no differences in age structure between the exclosures and the open woodland for either mice or voles, although this may be due to limited data concerning juveniles as they do not always trigger traps due to their weight.

The sex ratio was male biased in the exclosure sites for both mice and voles, although this pattern was not significant (mean proportion males in population, mice_ex_ = 0.65±0.04se, mice_ow_ = 0.43±0.09se, N = 3, W = 6, p = 0.11; voles_ex_ = 0.59±0.12se, voles_ow_ = 0.41±0.08se, N = 3, W = 5, p = 0. 29, Wilcoxon rank sum tests).

Reproductive activity peaked from July through to September for both mice and vole populations throughout the sites but was not significantly different between the exclosure and open woodland habitats (Mice: males, N = 3, W = 6, p = 0.11, females, N = 3. W = 3, p = 1; Voles: males, N = 3, W = 4, p = 0.59, females, N = 3, W = 4, p = 0.59, Wilcoxon rank sum tests).

Environment (when nested within site) was not a significant factor (Mice: F_3, 152_ = 1.50 p = 0.218, Voles: F_3, 148_ = 0.48, p = 0.697, ANOVA) when considering the weight of individuals in the exclosures compared to the open woodland (Mice: Mean adult weight, males_ex_ = 18.9±0.5se,, males_ow_ = 19.2±0.6se, females_ex_ = 17.4±0.5se, females _ow_ = 17.0±0.4se; Voles: Mean adult weight, males_ex_ = 18.8±0.5se, males_ow_ = 18.8±0.6se, females_ex_ = 17.0±0.9se, females_ow_ = 17.6±0.6se,).

### 4. Vegetation Survey

#### Ground flora

Species richness appeared to be higher along the open woodland transects at each site (mean = 12.3±0.7SE) compared to the exclosures (mean = 9.3±0.3SE) in the June surveys although this result was not significant (N = 3, W = 6, p = 1.11). There was some evidence that unpalatable, grazing tolerant grasses such as Tufted hair-grass (*Deschampsia cespitosa* L.) and False brome (*Brachypodium sylvaticum* (Huds) P. Beauv.), were found more often along the transects than in the exclosure at all sites (*Dc*_ex_/*Dc*_ow_ = 2, N = 3, W = 6, p = 0.10, Wilcoxon rank sum test). Unexpectedly, palatable species such as Bluebells (*H. non-scripta*), were also found more frequently in the open woodland (*Hs*_ex_/*Hs_ow_* = 7.7; N = 3, W = 6, p = 0.11, Wilcoxon rank sum test). The only species found consistently more frequently inside exclosures was Enchanter's nightshade (*Circaea lutetiana L*) (*Cl*_ex_/*Cl*_ow_ = 3.3; N = 3, W = 6, p = 0.11, Wilcoxon rank sum test).

#### Undergrowth

**Figure 4:**
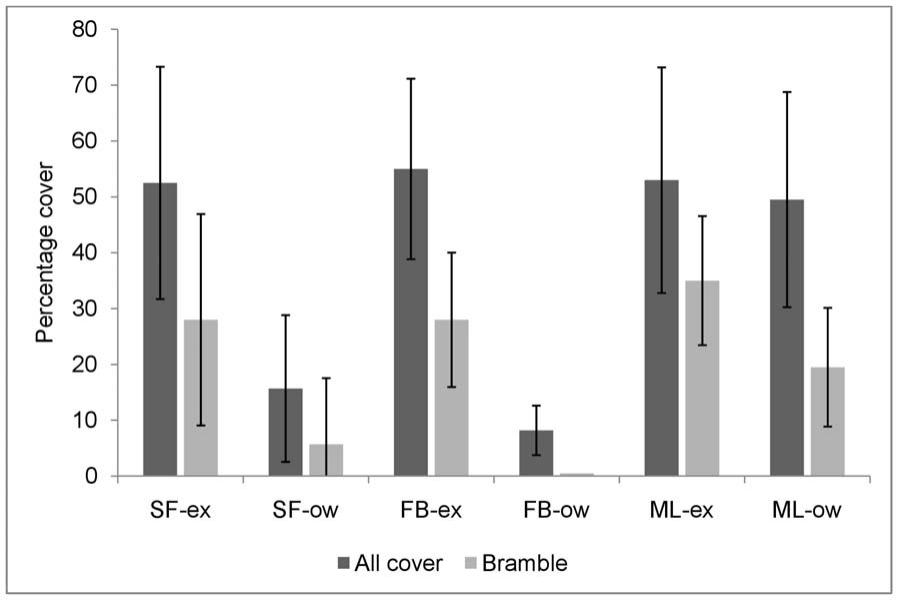
Percentage cover of the undergrowth layer. Sites: Swinford (SF),Firebreak (FB) and Marley (ML), Environments: Exclosure (-ex), Open woodland (-ow). Mean percentage shown (± standard error) for total undergrowth cover (including bramble, *Rubus fruticosus*) and bramble alone. Bramble cover has established a mean of 10% in the open woodland, but varies considerably according to site.

**Figure 5:**
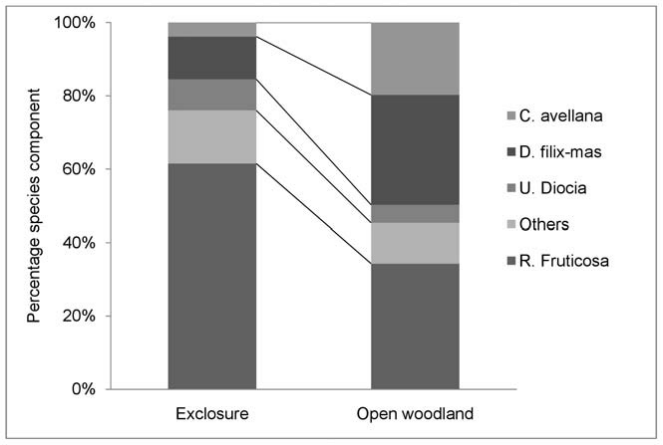
Composition of the undergrowth layer. Mean percentage cover of constituent plant species; *U.diocia* (Nettles), *C. Avellana* (Hazel), *D. Filix-mas* (Male fern), *R. Fruticosus* (Bramble). Bramble dominates the exclosures.

The percentage cover of plants in the undergrowth was much reduced in the open woodland for the sites SF and FB compared to the exclosure, but showed no difference for ML (Figure 4) (Undergrowth_ex_ = 53.5, undergrowth_ow_ = 24.5, N = 3, W = 6, p = 0.11, Wilcoxon rank sum test). There were also compositional differences between the exclosures and open woodland. The mean values showed a consistent pattern across all sites; Male fern (*Dryopteris filix-mas* (L.) Schott), and Hazel (*Corylus avellana* L.), made up a greater proportion of the vegetation in the undergrowth height zone in the open woodland, whereas bramble (*R. fruticosus*), nettles (*U. diocia*) and other species dominated in the exclosures (Figure 5). The fruiting index of bramble in the open woodland was low, with three times fewer floral apices observed per unit bramble cover than in the exclosures (fruit_ex_ = 0.42±0.05, fruit_ow_ = 0.14±0.08, N = 3, W = 6, p = 0.11, Wilcoxon rank sum test).

#### Canopy

The degree of canopy cover between open woodland and exclosure environments showed no consistent pattern (gap_exclosure_/gap_open woodland_ = 1.2, N = 3, W = 3, p = 1, Wilcoxon rank sum test). Site specific effects were observed, with canopy being the most closed at SF (gap = 0.12) compared to FB and ML (gap (same both sites) = 0.24). Hazel (*C. avellana*) and Ash (*Fraxinus excelsior* L.), were the major components of the canopy at all sites and both inside and outside the exclosures.

## Discussion

In this study, we have taken advantage of the rare opportunity to investigate an ecological question by experimentally manipulating a natural environment and documenting its recovery after a change in management. The literature concerning the effects of ungulates on woodlands has, until recently, mostly consisted of observations of ecosystem decline [1], [5], [9], [15]. The results of this study are therefore important alongside other exclosure studies (e.g. Bradfield Woods [13], [44] and the New Forest [16] in the UK, and ‘De Hoge Veluwe’ national park in the Netherlands [45]) to increase our understanding of the cascade effects of ungulate grazing in forest ecosystems, and the lag time between control of the cause and recovery of the woodland and the animal communities inhabiting it.

The studied deer exclosures constitute fragments within the forest itself, and as such, trapping along the perimeter of the area due to practical constraints (avoidance of trampling in favour of long-term vegetation monitoring) will have been subject to habitat edge effects. It is uncertain whether small mammals are responsive to edge effects and studies in forest patches have reached contrasting conclusions (e.g. Red-backed Voles [46], [47] and White-Footed Mice [41]).

In 2010, bank vole and wood mouse numbers appear to have recovered from the detrimental effects of heavy deer grazing in the 1990s. For mice, the best model incorporated random movement between the two environments, representing a homogeneous habitat for this species. The movement for voles was markovian and on average favored movement directed towards the exclosure although this was influenced mainly by FB where very few voles were sustained in the open woodland.

According to source/sink theory a source constitutes higher quality habitat for a species indicated by higher survival rate and reproductive rate and net emigration [48] compared to a sink or pseudo-sink which constitutes lower habitat quality which in the absence of a source cannot sustain a population (sink) or sustains a much reduced population (pseudo-sink) and is evidenced by lower abundance, survival and by net immigration [49].

Comparing apparent survival, probability of movement and abundance there is no evidence for a source/sink dynamic for wood mice between the deer-free exclosures and the open woodland. Comparing these vital rates for the bank voles there is some indication of variability between locations but this is not caused by the presence or absence of deer and probably describes a patchy and locally determined population throughout the woodland site. This constitutes a marked contrast to the 2001–2003 dataset [21] where bank voles were significantly more abundant in the exclosures than the open woodland and outnumbered wood mice in the exclosures (wm/bv = 0.23±0.06), whereas wood mice outnumbered bank voles in the open woodland (wm/bv = 1.6±0.52). Using the long-term small mammal data set for the woodland [22] as a reference, the mean ratio of mice to voles recorded in 2010 is comparable with that of the late 1980s early 1990s, before the peak in deer density and associated grazing pressure, although it can be seen from Table 3 that this ratio varies widely according to site. ML and FB show interspecific ratios more comparable with historic data from the 1960s and 70s whilst FB remains as an outlier and poor bank vole territory.

Population density and demographic ‘fitness’ parameters are often used to indicate the quality of a particular habitat type for a species, although in various studies of habitat gradients for small mammals (e.g. Deer mice and wild forest fires [26], White-footed mice and woodland-pasture mosaic [50]), individual fitness remains the same across a gradient of habitat suitability. At Wytham, we found no evidence for dispersal of young into the open woodland plots, or changes in reproductive activity or weight that would indicate a reduction in habitat suitability for either species.

Local factors, rather than presence or absence of deer, appear to affect bank vole, albeit not wood mouse numbers, possibly due to local cyclic effects based on transient factors such as the home range of a predator, population effects such as disease, or a locally failed food source, although none of these were evident from the data collected. A loose correlation was observed between undergrowth cover at the site and population size, mirroring previous results from these sites [21] and other studies [45], [51].

The decrease in bank vole population size and increasing abundance of wood mice observed in the long-term data set and the exclosure studies in 2001–2003 was attributed to the effects of grazing on woodland vegetation and subsequent differential habitat selection by the two species due to their respective survival strategies [15], [21], [22]. The return of bank vole numbers to earlier levels may thus in part be explained by recovery of the vegetation in the open woodland, through the effective management of deer numbers. In particular, the mean percentage cover of *R. futicosus* has increased from <1% along the open woodland transects in 2003 [21] to a mean of 10.1% in 2010, an observation which has been confirmed by an extensive study of the vegetation [6]. Bramble provides both food and cover, and we suggest that it constitutes a key species in determining bank vole populations in UK woodlands. The reproductive output for bramble, represented as a ‘fruiting index’, showed a significant reduction in the open woodland compared to the exclosures, suggesting young growth in open woodland as growing shoots only flower in their second year [52], and thus residual effects of deer grazing. Similar relationships have been observed in another related system in Sweden, where grazing by moose affects bilberry growth, with cascading effects for bank vole populations [53], [54].

We propose that it is the level of habitat complexity that determines small mammal population size. This has reached a threshold - the exact position of which we cannot identify, only to say that it has been achieved by management to maintain a deer density of <0.17 deer per hectare, over a time period of seven years - over which the small mammals, and particularly bank voles can maintain equivalent populations in both, the open woodland and the deer-free exclosures, despite some residual signs of disturbance. This corresponds with the successional response of small mammals to other causes of disturbance such as wild-fires and timber extraction in North American boreal forest [55], and wildfire in the coastal wet heath of New South Wales, Australia [56]. In these studies, the population recovery of small mammal species was dependent on vegetation density and complexity, not time elapsed since the disturbance. Small mammal communities respond rapidly to disturbances by changes in relative abundance of species adapted to different levels of habitat complexity and food availability.

We conclude that, due to their short generation spans, multiple litters within years and dispersal of young adults in the autumn to unoccupied sub-optimal territories, small mammal communities are able to recover relatively rapidly from the detrimental effects of disturbance such as intense deer grazing once the vegetative habitat has recovered past a certain threshold under favourable conditions. However, we caution that the potential for a source/sink dynamic may remain in recently disturbed environments between patches of old growth and the surrounding habitat, especially in harsh conditions such as cold winters or food shortages.
